# Vector-field dynamic x-ray (VF-DXR) using optical flow method in patients with chronic obstructive pulmonary disease

**DOI:** 10.1186/s41747-021-00254-w

**Published:** 2022-01-31

**Authors:** Takuya Hino, Akinori Tsunomori, Akinori Hata, Tomoyuki Hida, Yoshitake Yamada, Masako Ueyama, Tsutomu Yoneyama, Atsuko Kurosaki, Takeshi Kamitani, Kousei Ishigami, Takenori Fukumoto, Shoji Kudoh, Hiroto Hatabu

**Affiliations:** 1 Center for Pulmonary Functional Imaging, Department of Radiology, Brigham and Women’s Hospital and Harvard Medical School, 75 Francis Street, Boston, MA 02115 USA; 2 R&D Promotion Division, Healthcare Business Headquarters, Konica Minolta, Inc., 2970 Ishikawa-machi, Hachioji-shi, Tokyo, Japan; 3 Department of Radiology, Graduate School of Medicine, Osaka University, 2-2 Yamadaoka, Suita, Osaka, Japan; 4 Department of Clinical Radiology, Graduate School of Medical Sciences, Kyushu University, 3-1-1 Maidashi, Higashi-ku, Fukuoka, Fukuoka Japan; 5 Department of Diagnostic Radiology, Keio University School of Medicine, 35 Shinanomachi, Shinjuku-ku, Tokyo, Japan; 6 Department of Health Care, Fukujuji Hospital, Japan Anti-Tuberculosis Association, 3-1-24 Matsuyama, Kiyose, Tokyo, Japan; 7 Department of Diagnostic Radiology, Fukujuji Hospital, Japan Anti-Tuberculosis Association, 3-1-24 Matsuyama, Kiyose, Tokyo, Japan; 8 Japan Anti-Tuberculosis Association, 1-3-12 Kanda-Misakicho, Chiyoda-ku, Tokyo, Japan

**Keywords:** Lung, Optic flow, Pulmonary disease (chronic obstructive), Radiography (thoracic), Respiratory function tests

## Abstract

**Background:**

We assessed the difference in lung motion during inspiration/expiration between chronic obstructive pulmonary disease (COPD) patients and healthy volunteers using vector-field dynamic x-ray (VF-DXR) with optical flow method (OFM).

**Methods:**

We enrolled 36 COPD patients and 47 healthy volunteers, classified according to pulmonary function into: normal, COPD mild, and COPD severe. Contrast gradient was obtained from sequential dynamic x-ray (DXR) and converted to motion vector using OFM. VF-DXR images were created by projection of the vertical component of lung motion vectors onto DXR images. The maximum magnitude of lung motion vectors in tidal inspiration/expiration, forced inspiration/expiration were selected and defined as lung motion velocity (LMV). Correlations between LMV with demographics and pulmonary function and differences in LMV between COPD patients and healthy volunteers were investigated.

**Results:**

Negative correlations were confirmed between LMV and % forced expiratory volume in one second (%FEV_1_) in the tidal inspiration in the right lung (Spearman’s rank correlation coefficient, *r*_s_ = -0.47, *p* < 0.001) and the left lung (*r*_s_ = -0.32, *p* = 0.033). A positive correlation between LMV and %FEV_1_ in the tidal expiration was observed only in the right lung (*r*_s_ = 0.25, *p* = 0.024). LMVs among normal, COPD mild and COPD severe groups were different in the tidal respiration. COPD mild group showed a significantly larger magnitude of LMV compared with the normal group.

**Conclusions:**

In the tidal inspiration, the lung parenchyma moved faster in COPD patients compared with healthy volunteers. VF-DXR was feasible for the assessment of lung parenchyma using LMV.

**Supplementary Information:**

The online version contains supplementary material available at 10.1186/s41747-021-00254-w.

## Key points

• Lung motion velocity in tidal respiration showed a moderate correlation with percent predicted forced expiratory volume in one second (%FEV_1_).

• Chronic obstructive pulmonary disease (COPD) mild group had larger lung motion velocity than the normal group in tidal respiration.

• The lung parenchyma moved faster in COPD patients than in normal subjects.

## Background

Chronic obstructive pulmonary disease (COPD) is associated with increased mortality and comorbidity [1, 2]. It is widely accepted that the dysfunction of muscles as well as lung hyperinflation and chest wall remodeling causes the decreased mobility of the thorax [3]. Lung motion of normal subjects was assessed with four-dimension computed tomography (CT) applying robust feature matching, nonrigid point cloud registration, strain measurement, and sparse motion field method [4–7]. However, these studies did not completely reflect physiological lung motion because they were studied in the supine position, not in the standing position.

Recently, dynamic x-ray (DXR) using a flat panel detector with a large field of view was introduced. Previous studies have already shown the efficacy of DXR in measuring diaphragm or rib motion as well as projected lung area during a particular breathing phase in the standing position [8–13]. DXR enabled to obtain sequential images with high temporal resolution [14, 15]. The optical flow method (OFM) is one of the concentration gradient methods, providing visualization of moving objects by mathematical conversion of spatiotemporal concentration gradient to apparent motion [16]. This theory was based on three preconditions of movement between intervals as follows: unchanged luminance distribution, spatiotemporal differentiation, and minute movement. The OFM is applied to the recognition of moving objects or the quantification of movement in various situations [17–19]. Recent studies have demonstrated that physiological lung motion was potentially visualized with vector-field dynamic x-ray (VF-DXR) with sequential images of DXR using OFM [20]. During the spread of COVID-19, it may be helpful for this technique to provide the pulmonary functional data without physical contact between mouth and equipment. Even after the pandemic, the noncontact nature of this technique will be helpful against the possible emerging infection or novel respiratory diseases in the future. The absence of intrinsic circuit resistance is another unique characteristic of this new approach using dynamic x-ray.

We hypothesized that the altered motion of the lung parenchyma will be observed in patients with COPD when compared with that of healthy volunteers. The aim of this study is to assess the quantitative difference of dynamic lung motion between patients with COPD and healthy volunteers using VF-DXR with OFM.

## Methods

### Study population

This retrospective study was approved by the ethics committee and performed in accordance with the Declaration of Helsinki. The cohort was prospectively enrolled. Written informed consent was obtained from all the subjects prior to participation. From June 2009 to August 2011, consecutive COPD patients meeting the same inclusion criteria as those of previous studies were recruited [9, 11]: (1) clinical diagnosis of pure COPD based on clinical course, symptoms, chest CT examination, and pulmonary function test (PFT) with post-bronchodilator inhalation; (2) exclusion of other respiratory diseases such as acute respiratory infection, bronchiectasis, or any type of interstitial lung disease; (3) current or former smokers; (4) ≥ 20-year-old adults who gave informed consent, including for additional x-ray exposure within tolerant range compared to conventional chest radiography; (5) no status of pregnancy, potential pregnancy, or lactation; (6) scheduled for conventional chest radiograph; (7) ability to follow instructions for tidal or forced breathing. All the COPD subjects were classified into spirometry grades from 1 to 4 by global initiative for chronic obstructive lung disease (GOLD) with corresponding percent predicted forced expiratory volume in one second (%FEV_1_) [21]. COPD mild group was defined as GOLD 1 or 2 (*i.e.*, %FEV_1_ ≥ 50), and COPD severe group was defined as GOLD 3 or 4 (*i.e.*, %FEV_1_ < 50) in this study.

On the other hand, healthy volunteers as a control group were also recruited from May 2013 to February 2014. The following inclusion criteria were the same as those of previous studies [9, 11]: (1) ≥ 20-year-old adults who gave informed consent, including for additional x-ray exposure by DXR; (2) scheduled for conventional chest radiograph; (3) PFT results within normal limits, namely percent predicted vital capacity (%VC) > 80% and forced expiratory volume percent in one second divided by forced vital capacity (FEV_1_%) > 70%; (4) ability to follow instructions for forced or tidal breathing; (5) never smokers; (6) no status of pregnancy, potential pregnancy or lactation; (7) no past medical history of respiratory diseases.

The inclusion criteria were the same as those of previous studies [10, 12].

### Image acquisition

Posteroanterior view of chest DXR examination in standing position was performed with a prototype x-ray system (Konica Minolta Inc., Tokyo, Japan) composed of a flat panel detector (PaxScan 4030CB, Varian Medical Systems Inc., Salt Lake City, UT, USA) and a pulsed x-ray generator (DHF-155HII) with cineradiography option (Hitachi Medical Corporation, Tokyo, Japan). During the examination, COPD patients took several tidal breaths and one forced breath separately, while normal subjects took several tidal breaths, followed by one forced breath. Conditions for DXR examination were the same as the previous reports [9–13]: tube voltage 100 kVp; tube current 50 mA; pulse duration of pulsed x-ray 1.6 ms; source-to-image distance 2 m; additional filter 0.5 mm Al plus 0.1 mm Cu. The additional filter was used to filter out soft x-rays. The exposure time was approximately 10–15 s. The pixel size was 388 × 388 μm, the matrix size was 1024 × 768, and the overall image area was 40 × 30 cm. The dynamic image data, captured at 7.5–15 frames/s, were synchronized with the pulsed x-ray, which prevented excessive radiation exposure to the subjects. The entrance surface dose was approximately 0.3–1.0 mGy. Each DXR image was processed with bone suppression and converted into video with 5 frames/s before application of the optical flow method.

### Image conversion to video using OFM

Total variation regularization and robust L1 norm, TV-L1, optical flow estimation were adopted for motion analysis [22]. The rectangle-shaped region of interest (ROI) including the unilateral lung field in the forced inspiratory phase was located on both sides of the video. The size of the ROI of either side was usually different. Then, a larger ROI was set symmetrically with the same horizontal level as a substitute for a smaller ROI. Bilateral lung fields within the ROI were subdivided into 4-cm^2^ pixels to assess lung motion vector using OFM. In each pixel, the subtle interval difference in density between temporally successive DXR images with bone suppression was converted into motion vector using OFM implemented by OpenCV [23]. The obtained motion vectors were multiplied by five to represent motion/s. Only vertical motion vectors of all the pixels were superimposed on DXR images. The longest lung motion vector onto VF-DXR images in tidal inspiration, tidal expiration, forced inspiration, and forced expiration were extracted and defined as lung motion velocity (LMV). Downward direction of vectors was defined as positive direction.

### Data analysis

Demographic data among normal, COPD mild, and COPD severe groups were tested using one-way analysis of variance on ranks. The difference in sex ratio among the three groups was compared using Fisher’s exact test. Pulmonary function data such as tidal volume (TV), vital capacity (VC), %VC, FEV_1_, FEV_1_%, and %FEV_1_ were assessed with one-way analysis of variance on ranks. The association of LMV with demographic data or pulmonary function was analyzed with Spearman’s rank correlation coefficient. Scatter plots of %FEV_1_ *versus* LMV in each phase or side were visualized. The difference in LMV in both lungs in tidal and forced inspiratory and expiratory phases among three groups was analyzed with one-way analysis of variance on ranks. Post-hoc multiple comparisons were made using the Holm-Bonferroni method. A two-group comparison of LMV between normal subjects and COPD patients were also performed using the Mann-Whitney *U* test.

Statistical assessment was performed using R 4.0.3 (R Foundation for Statistical Computing, Vienna, Austria) with the EZR graphical user interface (Saitama Medical Center, Jichi Medical University, Saitama, Japan) [24, 25]. More precisely, EZR is a modified version of R commander designed to add statistical functions frequently used in biostatistics. In each test, a two-sided *p* value of less than 0.05 was considered statistically significant.

## Results

### Inclusion and exclusion criteria

**Fig. 1:**
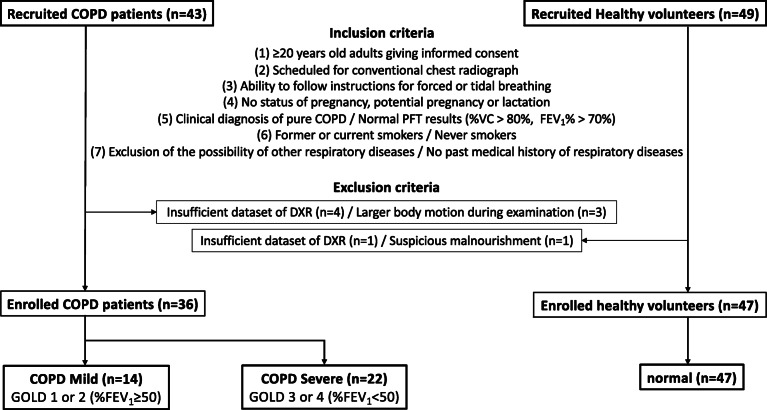
Flow chart of inclusion and exclusion criteria for this study. *COPD* Chronic obstructive pulmonary disease, *DXR* Dynamic x-ray, *FEV*_*1*_*%* Forced expiratory volume percent in one second divided by forced vital capacity, *GOLD* Global initiative for chronic obstructive lung disease, *PFT* Pulmonary function test, *%VC* Percent predicted vital capacity

The flow chart of inclusion and exclusion criteria are summarized in Fig. 1. Forty-three COPD patients as well as 49 healthy volunteers were meeting the inclusion criteria. Seven COPD patients and two healthy volunteers were excluded due to incomplete dataset, body motion, or suspicious malnourishment. Finally, a total of 36 COPD and 47 healthy subjects were analyzed in this study. Fourteen patients were classified as COPD mild, and 22 patients as COPD severe. Forty-seven healthy volunteers served as normal subjects, which is the same as those of the previous study [9].

### Demographic data and example cases

**Fig. 2:**
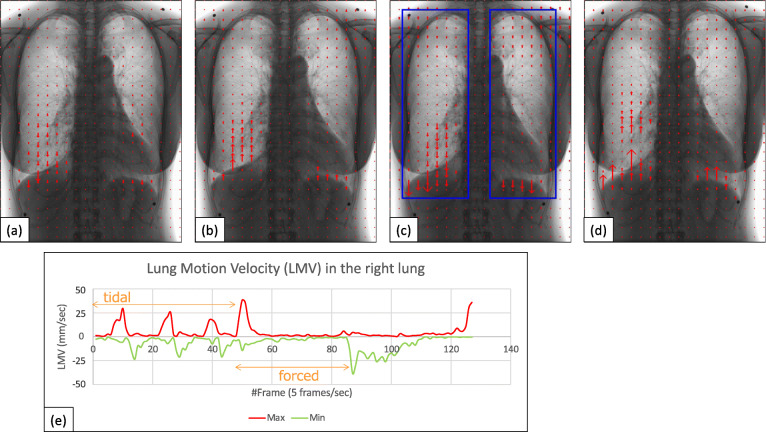
A 58-year-old male healthy volunteer with FEV_1_% 73.3 and %FEV_1_ 92.4, included in the normal group. VF-DXR images: (**a**) tidal inspiratory, (**b**) tidal expiratory, (**c**) forced inspiratory, and (**d**) forced expiratory phases. Blue rectangles in Fig. 1c represent ROIs in both lungs. The graph (**e**) shows the temporal change between LMV in the right lung and the number of frames. The frame rate is set to 5 frames/s (Video 1, Supplemental material). *FEV*_*1*_*%* Forced expiratory volume percent in one second divided by forced vital capacity, *%FEV*_*1*_ Percent predicted forced expiratory volume in one second, *LMV* Lung motion velocity, *VF-DXR* Vector-field dynamic x-ray, *ROI* Region of interest

**Fig. 3:**
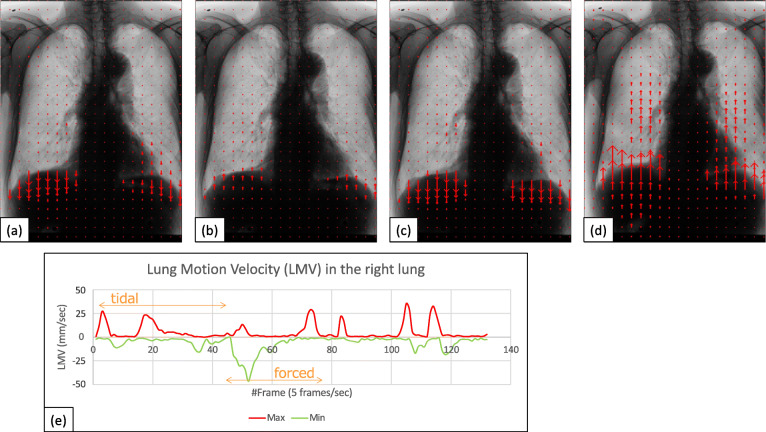
A 53-year-old made, COPD patient with FEV_1_% 60.3 and %FEV_1_ 79.6, which meets the criteria of GOLD 2, COPD mild group. VF-DXR images: (**a**) tidal inspiratory, (**b**) tidal expiratory, (**c**) forced inspiratory, and (**d**) forced expiratory phase. The graph (**e**) shows the temporal change between LMV in the right lung and the number of frames. The frame rate is set to 5 frames/s. *COPD* Chronic obstructive pulmonary disease, *FEV*_*1*_% Forced expiratory volume percent in one second divided by forced vital capacity, %*FEV*_*1*_ Percent predicted forced expiratory volume in one second, *GOLD* Global initiative for chronic obstructive lung disease, *LMV* Lung motion velocity, *VF-DXR* Vector-field dynamic x-ray

**Fig. 4:**
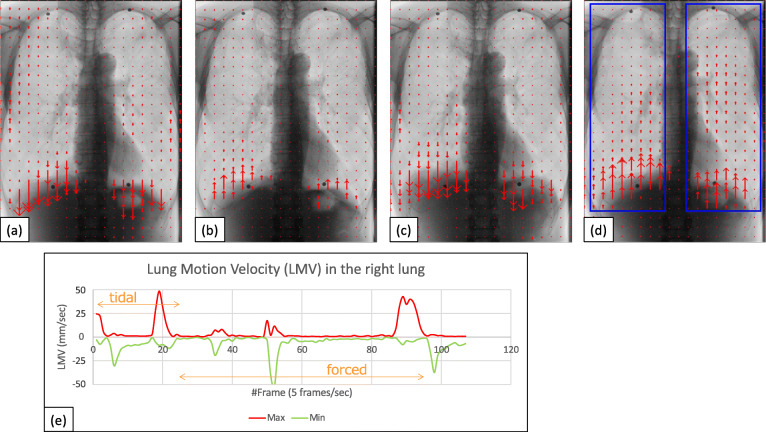
A 65-year-old male, COPD patient with FEV_1_% 38.5 and %FEV_1_ 16.3, which meets the criteria of GOLD 4, COPD Severe group. Blue rectangles in Fig. 3 (**d**) represent bilateral ROIs. VF-DXR images in (**a**) tidal inspiratory, (**b**) tidal expiratory, (**c**) forced inspiratory, and d forced expiratory phase. Blue rectangles represent bilateral ROIs. (**e**) The graph shows the temporal change between LMV in the right lung and the number of frames. The frame rate is set to 5 frames/s (Video 2 in the Supplemental material). *COPD* Chronic obstructive pulmonary disease, *FEV*_*1*_*%* Forced expiratory volume percent in one second divided by forced vital capacity, *%FEV*_*1*_ Percent predicted forced expiratory volume in one second, *GOLD* Global initiative for chronic obstructive lung disease, *LMV* Lung motion velocity, *ROI* region of interest, *VF-DXR* Vector-field dynamic x-ray

Demographic data and pulmonary function tests data among normal, COPD mild, and COPD severe groups were summarized in Table 1. Age, sex, and pulmonary function were different among the three groups with statistical significance (*p* < 0.001); COPD mild and severe groups mainly consisted of elderly males with a pulmonary functional disorder. The examples of VF-DXR images of the thorax during tidal and forced respiratory cycles are shown for normal (Fig. 2), COPD mild (Fig. 3), and COPD severe groups (Fig. 4). LMVs were largest in the lower lung fields in the tidal and forced inspiratory phases, and smallest in lower lung fields in the tidal and forced expiratory phases.

**Table 1:** Demographic data and PFTs among normal, COPD mild, and COPD severe groups

Variables	Normal (*n* = 47)	COPD mild (*n* = 14)	COPD severe (*n* = 22)	*p* value
	Median [IQR]	Median [IQR]	Median [IQR]	
Age (years)	55 [49, 61]	77.5 [71.5, 79]	67.5 [63.5, 76.3]	< 0.001
Sex, females/males	27/20	2/12	3/19	< 0.001
Height (cm)	162.3 [155, 169.6]	164.5 [161.5, 167]	164.5 [158.3, 167.5]	0.907
Weight (kg)	61.2 [51.6, 66.3]	57.9 [54.6, 62.5]	50.0 [47.0, 60.8]	0.210
BMI (kg/m^2^)	22.7 [20.4, 23.7]	22.6 [20.5, 23.4]	19.7 [18.0, 22.5]	0.083
Smoking History	Never	Current or former	Current or former	
GOLD, 1/2/3/4	Not applicable	4/10/0/0	0/0/17/5	
PFT				
TV (L)	0.60 [0.50, 0.98]	1.09 [0.75, 1.23]	0.84 [0.72, 0.97]	0.006
VC (L)	3.25 [2.64, 3.80]	3.11 [2.85, 3.50]	2.47 [1.94, 2.79]	< 0.001
%VC	106.1 [99.9, 117.5]	104.3 [91.5, 115.2]	79.9 [68.9, 90.4]	< 0.001
FEV_1_ (L)	2.58 [2.18, 3.19]	1.72 [1.56, 1.98]	0.93 [0.82, 1.03]	< 0.001
FEV_1_%	81.6 [79.0, 85.7]	58.8 [53.6, 60.7]	41.2 [36.6, 44.8]	< 0.001
%FEV_1_	105.2 [98.7, 113.8]	70.2 [59.1, 79.4]	37.2 [31.2, 42.4]	< 0.001

Demographic data including age, height, weight, and BMI were assessed with one-way analysis of variance. Sex distribution was evaluated with Fisher’s exact test. Each pulmonary functional data was examined with one-way analysis of variance on ranks. *BMI* Body mass index, *COPD* Chronic obstructive pulmonary disease, *FEV*_*1*_ Forced expiratory volume in one second; *FEV*_*1*_*%* Forced expiratory volume percent in one second divided by forced vital capacity, *%FEV*_*1*_ Percent predicted forced expiratory volume in one second; *GOLD* Global initiative for chronic obstructive lung disease; *IQR* Interquartile range, *PFT* Pulmonary function tests; *TV* Tidal volume, *VC* Vital capacity, *%VC* Percent predicted vital capacity

### The correlation of LMV with demographic data

**Fig. 5:**
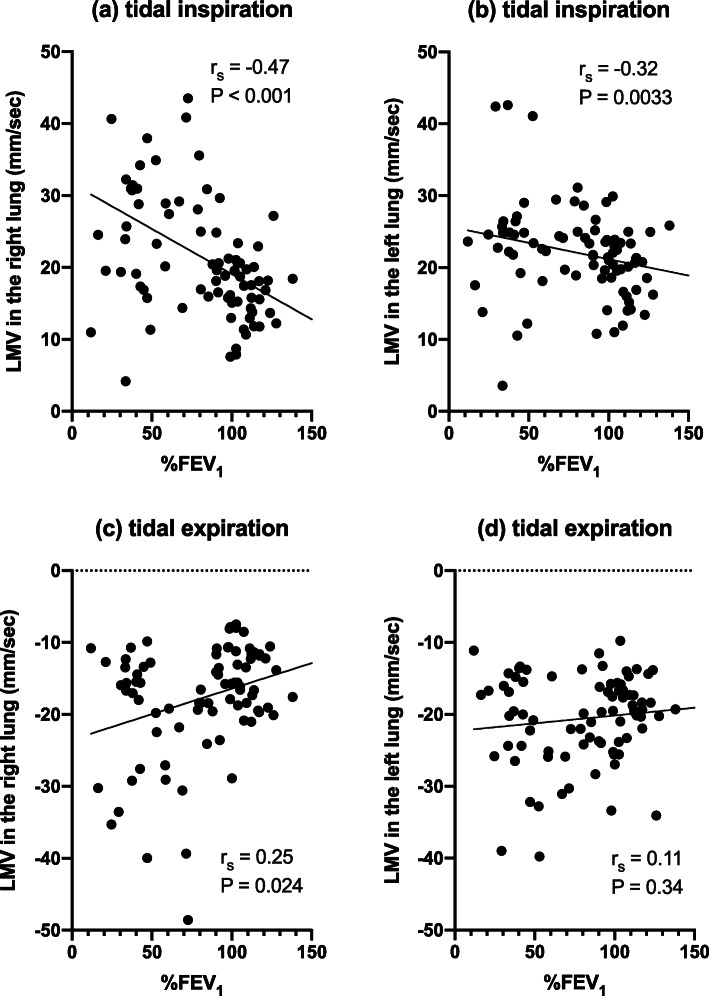
Scatter plots of %FEV_1_ and bilateral LMV in tidal respiratory phase with approximate line. LMV in the tidal inspiration phase *versus* %FEV_1_ in the right lung (**a**) and in the left lung (**b**). LMV in the tidal expiration phase *versus* %FEV_1_ in the right lung (**c**) and in the left lung (**d**). In each phase, the statistically significant correlation between %FEV_1_ and LMV in tidal inspiration in both lungs as well as those between %FEV_1_ and LMV in tidal expiration in the right lung was indicated by the Spearman rank correlation coefficient. *COPD* Chronic obstructive pulmonary disease, *%FEV*_*1*_ Percent predicted forced expiratory volume in one second, *LMV* Lung motion velocity, *r*_*s*_ Spearman’s rank correlation coefficient

Scatter plots with LMV *versus* %FEV_1_ in the tidal inspiratory and expiratory phases were shown in Fig. 5. There were significant negative correlations between LMV and %FEV_1_ in the tidal inspiratory phase in both lungs (right, *r*_s_ = -0.47, *p* < 0.001; left, *r*_s_ = -0.32, *p* = 0.003). A significant positive correlation between LMV and %FEV_1_ in the tidal expiratory phase was observed in the right lung (*r*_s_ = 0.25, *p* = 0.024), but not in the left lung. Tables 2 and 3 demonstrate the degree of correlations between LMV and demographic data and pulmonary function tests in tidal and forced inspiratory and expiratory phases in the right lung (Table 2) and in the left lung (Table 3). Mild to moderate correlation between LMV and %FEV_1_ was observed in both lungs in the tidal inspiratory and expiratory phases. LMV in the tidal inspiratory and expiratory phases in the right lung also showed mild to moderate correlation with FEV_1_, FEV_1_%, and age (Tables 2 and 3).

**Table 2:** Correlation between lung motion velocity in the right lung and demographic data

Variables	Tidal inspiration		Tidal expiration		Forced inspiration		Forced expiration	
	r_s_ [95% CI]	*p* value	r_s_ [95% CI]	*p* value	r_s_ [95% CI]	*p* value	r_s_ [95% CI]	*p* value
Age	0.34 [0.13, 0.52]	0.002^**^	-0.32 [-0.51, -0.11]	0.003^**^	0.09 [-0.13, 0.31]	0.404	-0.27 [-0.46, -0.05]	0.015^*^
Sex	0.20 [-0.02, 0.41]	0.068	-0.08 [-0.30, 0.14]	0.446	0.11 [-0.11, 0.33]	0.315	-0.25 [-0.45, -0.03]	0.023^*^
Height	0.07 [-0.15, 0.29]	0.500	-0.04 [-0.26, 0.18]	0.719	0.08 [-0.14, 0.30]	0.450	-0.30 [-0.49, -0.08]	0.007^**^
Weight	0.06 [-0.16, 0.28]	0.571	-0.05 [-0.27, 0.17]	0.656	0.05 [-0.17, 0.27]	0.657	-0.26 [-0.46, -0.04]	0.016^*^
BMI	0.06 [-0.17, -0.28]	0.595	-0.07 [-0.28, 0.16]	0.552	0.05 [-0.18, 0.27]	0.679	-0.16 [-0.37, 0.07]	0.157
TV	0.14 [-0.08, 0.35]	0.198	-0.17 [-0.38, 0.05]	0.120	0.16 [-0.06, 0.37]	0.144	-0.27 [-0.46, -0.05]	0.015^*^
VC	-0.19 [-0.40, 0.03]	0.073	0.12 [-0.10, 0.33]	0.278	-0.03 [-0.25, 0.19]	0.764	-0.14 [-0.35, 0.08]	0.194
%VC	-0.26 [-0.46, -0.04]	0.017^*^	0.11 [-0.11, 0.33]	0.309	-0.05 [-0.27, 0.18]	0.689	-0.05 [-0.27, 0.18]	0.664
FEV_1_	-0.40 [-0.57, -0.20]	<0.001^**^	0.26 [0.05, 0.46]	0.016^*^	-0.08 [-0.30, 0.18]	0.448	0.08 [-0.14, 0.30]	0.454
FEV_1_%	-0.42 [-0.59, -0.22]	<0.001^**^	0.25 [0.03, 0.45]	0.021^*^	-0.16 [-0.37, -0.06]	0.146	0.25 [0.03, 0.44]	0.025^*^
%FEV_1_	-0.47 [-0.63, -0.28]	<0.001^**^	0.25 [0.03, 0.45]	0.024^**^	-0.10 [-0.31, 0.12]	0.385	0.18 [-0.05, 0.25]	0.114

Spearman’s rank correlation coefficient (*r*_s_) was calculated. *p < 0.05; **p < 0.01. *BMI* Body mass index, *CI* Confidence interval, *FEV*_*1*_ Forced expiratory volume in one second, *FEV*_*1*_*%* Forced expiratory volume percent in one second divided by forced vital capacity, *%FEV*_*1*_ Percent predicted forced expiratory volume in one second, *GOLD* Global initiative for chronic obstructive lung disease, *LMV* Lung motion velocity, *TV* Tidal volume, *VC* Vital capacity; *%VC* Percent predicted vital capacity

**Table 3:** Correlation between lung motion velocity in the left lung and demographic data

Variables	Tidal inspiration		Tidal expiration		Forced inspiration		Forced expiration	
	r_s_ [95% CI]	*p* value	r_s_ [95% CI]	*p* value	r_s_ [95% CI]	*p* value	r_s_ [95% CI]	*p* value
Age	0.14 [-0.08, 0.35]	0.199	-0.17 [-0.37, 0.06]	0.135	-0.02 [-0.24, 0.20]	0.852	-0.19 [-0.40, 0.03]	0.079
Sex	0.27 [0.05, 0.47]	0.013^*^	-0.10 [-0.32, 0.12]	0.348	0.18 [-0.05, 0.38]	0.110	-0.27 [-0.46, -0.05]	0.013^*^
Height	0.08 [-0.15, 0.29]	0.500	-0.06 [-0.27, 0.17]	0.612	0.17 [-0.05, 0.38]	0.121	-0.31 [-0.50, -0.10]	0.004^**^
Weight	0.07 [-0.16, 0.29]	0.544	-0.05 [-0.26, 0.18]	0.681	0.22 [0, 0.42]	0.047^*^	-0.30 [-0.49, 0.08]	0.006^**^
BMI	0.05 [-0.17, 0.27]	0.628	-0.04 [-0.26, 0.18]	0.695	0.17 [-0.06, 0.38]	0.131	-0.20 [-0.40, 0.02]	0.072
TV	-0.05 [-0.27, 0.18]	0.663	0.05 [-0.18, 0.27]	0.667	0.13 [-0.09, 0.34]	0.232	-0.25 [-0.45, -0.03]	0.023^*^
VC	-0.03 [-0.25, 0.20]	0.807	0.06 [-0.17, 0.28]	0.595	0.21 [-0.01, 0.42]	0.053	-0.23 [-0.43, -0.01]	0.038^*^
%VC	-0.23 [-0.43, -0.01]	0.038^*^	0.12 [-0.10, 0.34]	0.261	0.15 [-0.08, 0.36]	0.186	-0.09 [-0.30, 0.14]	0.436
FEV_1_	-0.16 [-0.37, 0.06]	0.137	0.07 [-0.16, 0.28]	0.559	0.20 [-0.20, 0.41]	0.067	-0.06 [-0.28, 0.17]	0.613
FEV_1_%	-0.21 [-0.42, 0.01]	0.052	0.02 [-0.20, 0.24]	0.858	0.09 [-0.13, 0.31]	0.405	0.08 [-0.14, 0.30]	0.446
%FEV_1_	-0.32 [-0.51, -0.10]	0.003^**^	0.11 [-0.11, 0.32]	0.338	0.13 [-0.10, 0.34]	0.245	0.10 [-0.13, 0.31]	0.396

Spearman’s rank correlation coefficient (*r*_s_) was calculated. *p < 0.05; **p < 0.01. *BMI* Body mass index, *CI* Confidence interval, *FEV*_*1*_ Forced expiratory volume in one second, *FEV*_*1*_*%* Forced expiratory volume percent in one second divided by forced vital capacity, *%FEV*_*1*_ Percent predicted forced expiratory volume in one second, *GOLD* Global initiative for chronic obstructive lung disease, *LMV* Lung motion velocity, *TV* Tidal volume, *VC* Vital capacity; *%VC* Percent predicted vital capacity

### Difference in LMV among three groups

**Fig. 6:**
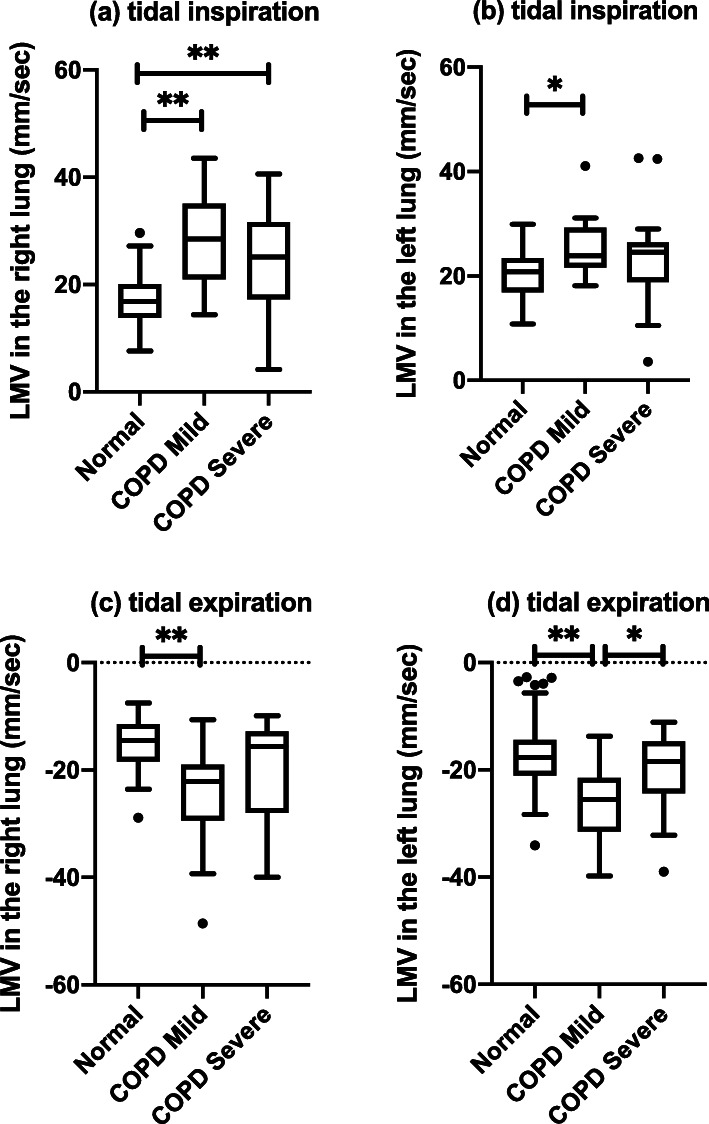
Box-and-whisker plots of bilateral LMV of normal, COPD mild, and COPD severe groups in the tidal respiration. In each phase, differences between groups were investigated using one-way analysis of variance on ranks. Multiple comparisons showed that the absolute value of LMV of the COPD mild group was larger than that of the normal group with statistical significance. *p < 0.05; **p < 0.01. *COPD* Chronic obstructive pulmonary disease, *LMV* Lung motion velocity

**Fig. 7:**
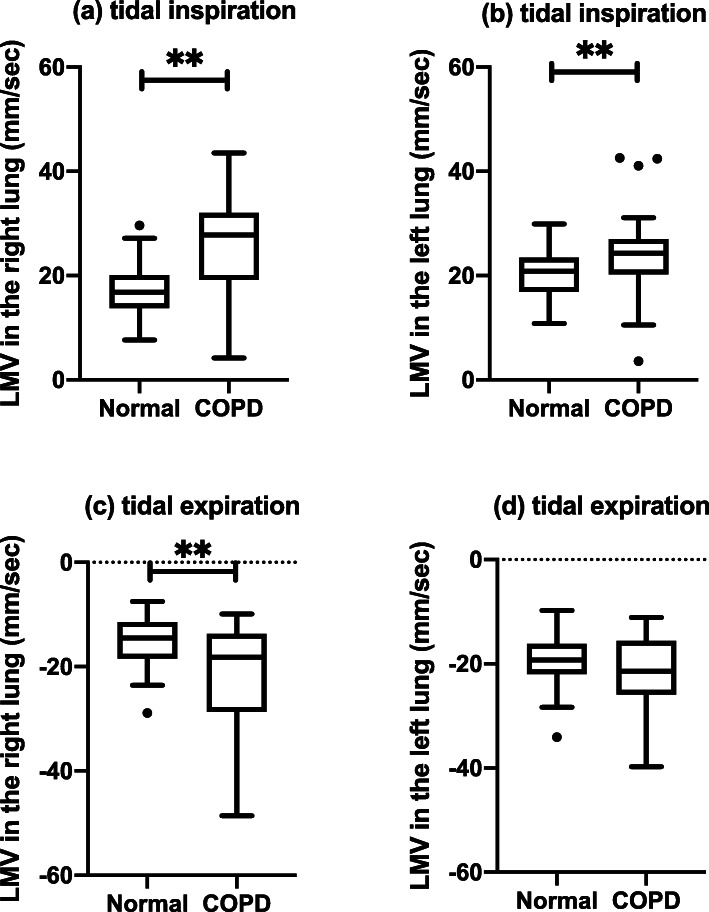
Box-and-whisker plots of bilateral LMV of normal and COPD groups in tidal respiration. The lung parenchyma moved faster in COPD patients compared with normal subjects in the tidal respiration in both lungs except for the tidal expiration in the left lung. **p < 0.01. *COPD* Chronic obstructive pulmonary disease, *LMV* Lung motion velocity

LMVs among normal, COPD mild, and COPD severe groups were summarized in Table 4. LMVs among groups were different in the tidal inspiratory and expiratory phases, which was confirmed by one-way analysis of variance on ranks. Post-hoc multiple comparison analyses with the Holm-Bonferroni method showed that the COPD mild group has a significantly larger magnitude of LMV compared with the normal group (Fig. 6). A similar trend was also observed in COPD severe group compared to the normal group, while the statistical significance was confirmed in only LMV in tidal inspiration in the right lung (Fig. 6). In two-group comparisons, the lung parenchyma moved faster in COPD patients compared with normal subjects in the tidal respiration in both lungs (*p* < 0.004) except for the tidal expiration in the left lung (Fig. 7).

**Table 4:** Lung motion velocity in tidal and forced inspiration and expiration phases among normal, chronic obstructive pulmonary disease (COPD) mild, and COPD severe groups

	Lung motion velocity, median [interquartile range] (mm/s)			*p* values			
	(1) Normal (*n* = 47)	(2) COPD mild (*n* = 14)	(3) COPD severe (*n* = 22)	All^a^	(1) vs (2) ^b^	(2) vs (3) ^c^	(1) vs (3) ^d^
Right							
Tidal inspiration	16.8 [14.1, 19.9]	28.5 [21.7, 33.9]	25.1 [17.8, 31.3]	< 0.001**	< 0.001**	0.432	0.002**
Tidal expiration	-14.5 [-18.4, -11.5]	-22.1 [-28.6, -19.2]	-15.6 [-25.2, -12.9]	< 0.001**	< 0.001**	0.059	0.206
Forced inspiration	28.8 [22.5, 36.2]	24.1 [20.7, 37.3]	36.3 [25.3, 47.6]	0.107	0.502	0.214	0.214
Forced expiration	-23.6 [-31.4, -18.5]	-32.9 [-46.6, -20.8]	-27.0 [-40.3, -21.9]	0.093	0.227	0.810	0.227
Left							
Tidal inspiration	20.8 [17.7, 23.4]	23.9 [22.3, 29.1]	24.6 [19.9, 26.2]	0.012*	0.018*	0.642	0.089
Tidal expiration	-19.3 [-21.5, -16.4]	-25.5 [-30.9, -22.0]	-18.4 [-23.8, -15.0]	0.009**	0.004**	0.038*	0.934
Forced inspiration	31.2 [27.4, 36.5]	28.3 [23.5, 38.6]	31.6 [26.1, 39.0]	0.678	1.000	1.000	1.000
Forced expiration	-27.1 [-33.5, -22.0]	-33.8 [-43.6, -27.7]	-27.8 [-39.0, -21.1]	0.171	0.166	0.479	0.650

Lung motion velocity downward is defined as positive direction. *p value ≤ 0.05. **p value ≤ 0.01. ^a^*p* values are for the comparison of all groups by one-way analysis of variance on ranks. Post-hoc multiple comparison was analyzed with Holm-Bonferroni method. ^b^*p* values are for the comparison between normal and COPD mild groups. ^c^*p* values are for the comparison between COPD mild and severe groups. ^d^*p* values are for the comparison between normal and COPD severe groups

## Discussion

This paper first demonstrated the quantitative assessment of lung motion in the standing position, using DXR with OFM. OFM was considered optimal to visualize lung motion due to the concentration gradient calculated from sequential DXR images with high spatiotemporal resolution. The current study demonstrated that VF-DXR with OFM is applicable to clinical research. Previous studies have reported the difference of pixel value change or diaphragm motion between normal subjects and COPD patients with DXR [8, 9]. The lung moves rhythmically in cooperation with the diaphragm and thoracic cage. It was expected that the lung motion between normal subjects and COPD patients is different.

Tanaka et al. [14] have reported DXR had the potential to visualize vector-field of lung motion using the cross-correlation method. OFM was initially proposed as one of the markerless tracking methods for lung tumors in chest radiographs [26]. OFM can visualize motion velocity with higher spatial resolution than the cross-correlation method [27]. Ichiji et al. [28] first applied OFM to sequential x-ray images to track the real-time motion of lung tumors, although the lung parenchymal motion itself was not studied in the paper. Recently, the feasibility of VF-DXR with OFM for lung parenchyma motion analysis was reported [21]. However, quantification of the motion vector of VF-DXR has not been assessed yet.

In this study, COPD patients showed larger LMV in tidal inspiration, compared with normal subjects. Peripheral muscle atrophy and dysfunction can cause hyperinflation followed by abnormal position or movement of the lower rib cage [29]. These mechanisms might induce larger LMV. Hyperinflation of the lungs, followed by a flattened diaphragm, might also contribute to larger LMV in COPD patients. The laterality of LMV may attribute to the lower level of the left diaphragm; the left lower lung field can move longer or faster, whereas the right diaphragm is oppressed by the liver. Heart or stomach gas are also possible causes of laterality.

Koyama et al. [30] assessed three-dimensional lung motion of COPD patients obtained by thin-sliced inspiratory and expiratory CT images in the supine position. They focused on the non-rigid registration model to visualize 3D motion vectors. They showed that craniocaudal lung motion was correlated with %FEV_1_ in COPD patients. On the other hand, VF-DXR adopted OFM as a simpler technique. VF-DXR is performed in the standing position without lateral images. VF-DXR can collect sequential images of continuous respiratory phases. VF-DXR in the standing position may be able to represent dynamic and physiological respiration more appropriately than CT. The lower radiation exposure compared with CT is another advantage of VF-DXR. The efficacy of OFM toward respiratory diseases other than COPD has not been proved yet. However, it has the potential to make the application for motion analysis of interstitial pneumonia or postoperative adhesion.

This study has several limitations. First, it has a small sample size: only 47 normal and 36 COPD subjects were analyzed. Reassessment with larger cases will be required to reconfirm the results of this study. Second, we focused on only the maximum vector in some phases, which did not perfectly reproduce the real lung motion or LMV. Third, this study depended on only the visual assessment of videos. It is also a problem that VF-DXR could not be compared with actual lung motion. Fourth, the ventrodorsal motion could not be analyzed owing to the lack of lateral image data in COPD patients.

In conclusion, the lung parenchyma moved faster in COPD patients compared with normal subjects in the tidal respiration. Significant negative correlations were observed between LMV and %FEV_1_ in the tidal inspiratory phase in both lungs. VF-DXR was feasible for the assessment of lung parenchyma in patients with COPD.

## Supplementary Information

**Additional file 1.** Videos of VF-DXR of Normal patient is shown in Videos 1. This case corresponds to Fig. 1.

**Additional file 2.** Videos of VF-DXR of COPD patient is shown in Videos 2. This case corresponds to Fig. 3.

## Data Availability

The data that support the findings of this study are available from Konica Minolta Inc. but restrictions apply to the availability of these data, which were used under license for the current study, and so are not publicly available. Data are however available from the authors upon reasonable request and with permission of Konica Minolta Inc.

## Abbreviations

%FEV_1_: Percent predicted forced expiratory volume in one second
%VC: Percent predicted vital capacity
COPD: Chronic obstructive pulmonary disease
CT: Computed tomography
DXR: Dynamic x-ray
FEV_1_%: Forced expiratory volume percent in one second divided by forced vital capacity
FEV_1_: Forced expiratory volume in one second
GOLD: Global initiative for chronic obstructive lung disease
LMV: Lung motion velocity
OFM: Optical flow method
PFT: Pulmonary function test
ROI: Region of interest
*r*_s_: Spearman’s rank correlation coefficient
TV: Tidal volume
VC: Vital capacity
VF-DXR: Vector-field dynamic x-ray
